# Tuberculosis-triggered cytokine storm with hemophagocytic lymphohistiocytosis and tuberculous spondylitis in an apparently immunocompetent host: a case report and literature review

**DOI:** 10.3389/fimmu.2025.1695605

**Published:** 2025-11-21

**Authors:** Liya Zhu, Jinzhi Lu, Yanfei Chen

**Affiliations:** 1 Department of Infectious Diseases, The First Affiliated Hospital of Yangtze University, Jingzhou, Hubei, China; 2 Department of Laboratory Medicine, The First Affiliated Hospital of Yangtze University, Jingzhou, Hubei, China; 3 Department of Infectious Diseases, The First Affiliated Hospital, College of Medicine, Zhejiang University School of Medicine, Hangzhou, Zhejiang, China

**Keywords:** hemophagocytic lymphohistiocytosis, tuberculous spondylitis, disseminated pulmonary tuberculosis, case report, hyperinflammation, cytokine storm, immunosenescence

## Abstract

Hemophagocytic lymphohistiocytosis (HLH) secondary to disseminated tuberculosis (TB) is a rare, life-threatening hyperinflammatory syndrome. We present a 60-year-old man with recurrent fever and syncope. Workup revealed cytopenias, hyperferritinemia (peak 5,802 ng/mL), elevated C-reactive protein (CRP), and hepatic dysfunction, fulfilling HLH-2004 criteria. Imaging showed miliary lung nodules and tuberculous spondylitis at T9. Bone marrow biopsy confirmed hemophagocytosis, and next-generation sequencing identified Mycobacterium tuberculosis. This case demonstrates that disseminated TB can trigger a fulminant cytokine storm even in an elderly host without overt immunodeficiency. Successful outcomes require combined antitubercular and immunomodulatory therapy.

## 1 Introduction

Pulmonary tuberculosis (TB) remains a global health threat. Its hematogenous dissemination can lead to serious conditions like tuberculous spondylitis (Pott disease) (1) and hemophagocytic lymphohistiocytosis (HLH), a hyperinflammatory syndrome often triggered by severe infection (2, 3). Diagnosing these entities is challenging due to their non-specific presentations, frequently leading to detrimental delays. We report a case of disseminated TB (miliary pulmonary and spinal) complicated by HLH in an elderly, apparently immunocompetent host. This case highlights the potential for mycobacterial infection to provoke a fulminant cytokine storm, underscoring the critical need for combined antimicrobial and immunomodulatory therapy.

## 2 Case presentation

**Figure 1:**
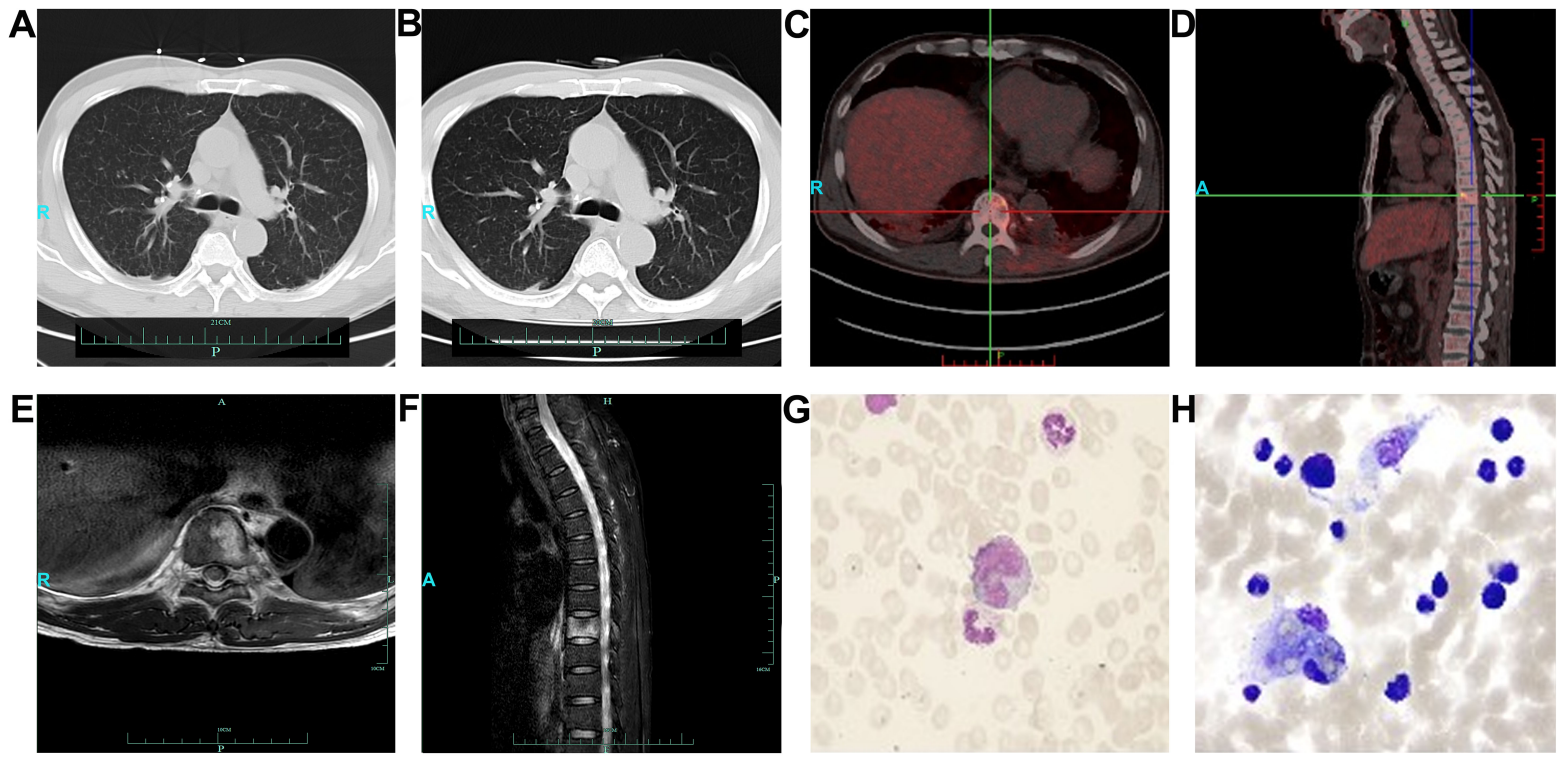
Disseminated tuberculosis with secondary hemophagocytic lymphohistiocytosis (HLH). **(A)** Initial chest CT (12 May) shows diffuse miliary opacities and pleural effusions. **(B)** Follow-up CT (24 May) demonstrates partial resolution of nodules. **(C, D)** 18F-FDG PET/CT (19 May) reveals a hypermetabolic T9 vertebral lesion (SUVmax 8.4), inflammatory foci, and hepatosplenomegaly. **(E, F)** Thoracic spine MRI (21 May) confirms T9 spondylitis with paraspinal edema and a prevertebral abscess. **(G, H)** Bone marrow aspirate (18 May) shows hypercellularity with hemophagocytosis (Wright-Giemsa stain, ×400). CT, computed tomography; FDG, fluorodeoxyglucose; MRI, magnetic resonance imaging; PET, positron emission tomography; SUVmax, maximum standardized uptake value.

A 60-year-old man presented with 14 days of recurrent fever (peak 39.2°C) and syncope. His history included hypertension, penicillin allergy, and a 20 pack-year smoking history. Notably, no history of tuberculosis contact or travel to TB-endemic areas was elicited. Despite empiric omadacycline for suspected pneumonia, he remained febrile and hypotensive (80/40 mmHg), requiring vasopressor support. Examination showed icteric sclera and skin without lymphadenopathy or hepatosplenomegaly. Admission labs revealed pancytopenia (WBC 1.75×10^9^/L, ALC 0.12×10^9^/L, platelets 71×10^9^/L), cholestatic hepatitis (bilirubin 86.9 μmol/L, ALT 170 U/L, AST 184 U/L), acute kidney injury (SCrea 126 μmol/L), and elevated inflammatory markers (CRP 119.5 mg/L, ferritin 2,521 ng/mL). Septic shock was evidenced by lactic acidosis (LAC 3.3 mmol/L) with compensatory respiratory alkalosis (pO_2_ 76.7 mmHg, pCO_2_ 26.5 mmHg). Serologies for common pathogens were negative, as was an initial T-SPOT.TB. However, chest CT demonstrated miliary nodules (**Figure 1A**), highly suggestive of disseminated tuberculosis, despite negative sputum and blood cultures and a bone marrow AFB stain.

### 2.1 Course in hospital

**Figure 2:**
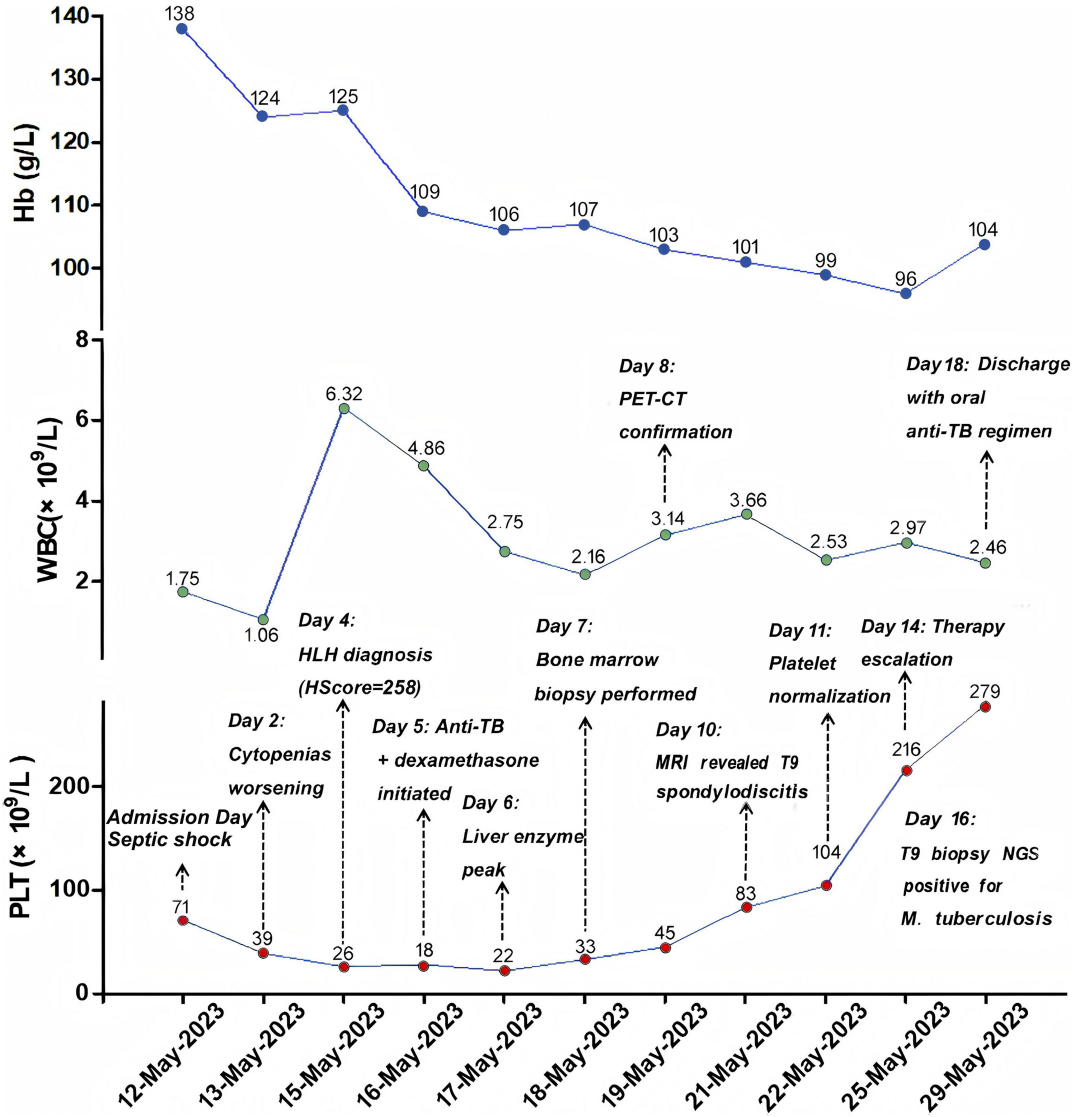
Temporal hematologic profile. Dynamic changes in hemoglobin (Hb; blue), platelet count (red), and white blood cell count (WBC; green) over time. Critical clinical events are annotated at corresponding time points. Hb, Hemoglobin; WBC, White Blood Cell.

Over the first five days, multi-organ dysfunction worsened (SOFA=6) with progressive cytopenias (platelet nadir 18×10^9^/L; **Figure 2**). He met HLH-2004 criteria with hypertriglyceridemia (4.1 mmol/L) and hypofibrinogenemia (1.2 g/L). Inflammatory markers surged (CRP 152.3 mg/L, ferritin 5,802 ng/mL), accompanied by worsening cholestatic hepatitis (**Table 1**). Day 4 immunology showed elevated cytokines (IL-6 22.47, IFN-γ 10.5 pg/mL) and profound CD4^+^ depletion (213 cells/μL). An HScore of 258 indicated >99% probability of HLH (**Supplementary Table S1**) (4). Following a positive repeat T-SPOT.TB on day 4, a dual-pathway strategy was initiated on day 5 with liver-sparing anti-TB therapy (moxifloxacin, amikacin) and immunomodulation (dexamethasone, IVIG) for HLH (**Table 2**).

**Table 1:** Serial laboratory parameters during hospitalization.

Date (hospital day)	CRP (mg/L)	Ferritin (ng/mL)	RBC (×10^12^/L)	ALC (×10^9^/L)	ALB (g/L)	ALT (U/L)	AST (U/L)	LDH (U/L)	T-Bil (μmol/L)	DB (μmol/L)	SCrea (μmol/L)
12 May 2023 (Day 1)	119.5	2521	4.25	0.12	33.8	170.1	184.2	821	86.9	73.6	126
13 May 2023 (Day 2)	152.3	4487	4.12	0.20	28.3	195.2	213.4	1502	90.5	77.5	122
15 May 2023 (Day 4)	125.3	5802	4.17	0.87	29.8	117.4	70.2	2213	95.6	91.3	100
16 May 2023 (Day 5)	115.6	5601	3.68	0.58	28.4	205.3	185.5	2806	96.8	91.5	95
17 May 2023 (Day 6)	85.1	5211	3.69	0.40	26.5	206.6	184.1	3019	97.2	91.5	91
18 May 2023 (Day 7)	65.6	4721	3.61	0.47	29.8	185.1	165.6	2502	92.1	86.2	90
19 May 2023 (Day 8)	45.5	4015	3.52	0.96	31.2	160.2	140.5	1817	82.3	77.5	89
21 May 2023 (Day 10)	28.2	2712	3.46	1.41	34.5	115.2	100.1	987	65.2	60.8	84
22 May 2023 (Day 11)	18.3	1908	3.38	1.01	36.7	85.8	70.7	605	48.7	43.1	72
25 May 2023 (Day 14)	9.5	951	3.27	1.10	37.2	60.1	45.5	351	32.3	28.2	74
29 May 2023 (Day 18)	5.5	452	3.50	1.09	38.4	42.3	32.1	262	21.5	17.5	78

Reference ranges: WBC, 4.0-10.0 ×10^9^/L; PLT, 125-350 ×10^9^/L; Hb, 130–175 g/L; RBC, 4.09-5.74 ×10^12^/L; ALC, 0.8-4.0 ×10^9^/L; CRP, 0–5 mg/L; ALB, 40–55 g/L; Ferritin, 15–200 ng/mL; ALT, 0–40 U/L; AST, 0–40 U/L; LDH, 120–250 U/L; T-Bil, 3.4-20.5 μmol/L; DB, 0-6.8 μmol/L; SCrea, 57-111 μmol/L.

WBC, white blood cell count; PLT, platelet count; Hb, hemoglobin; RBC, red blood cell count; ALC, absolute lymphocyte count; CRP, C-reactive protein; ALB, albumin; Ferritin, ferritin; ALT, alanine aminotransferase; AST, aspartate aminotransferase; LDH, lactate dehydrogenase; T-Bil, total bilirubin; DB, direct bilirubin; SCrea, Serum Creatinine.

**Table 2:** Pharmacotherapy summary.

Category	Agent	Dosing regimen	Duration	Purpose
Empiric Antibiotics	Omadacycline	0.1 g daily	12–15 May 2023	Broad-spectrum coverage for suspected atypical pneumonia
	Meropenem	1.0 g three times daily	16–28 May 2023	Empirical sepsis coverage
Anti-TB Therapy	Moxifloxacin	0.4 g daily	From 16 May 2023	Initial liver-sparing therapy (part of parenteral regimen)
	Amikacin	400 mg daily	18–24 May 2023	Initial liver-sparing therapy (8 mg/kg for 50 kg patient)
	Isoniazid (INH)	0.3 g daily	From 25 May 2023	First-line TB treatment
	Rifampicin (RIF)	0.45 g daily	From 25 May 2023	First-line TB treatment
	Ethambutol (EMB)	0.75 g daily	From 25 May 2023	First-line TB treatment
	Linezolid (LZD)	0.6 g twice daily	From 25 May 2023	Enhanced spinal TB penetration
HLH Management	Dexamethasone (DEX)	5 mg daily	16–22 May 2023	Immunosuppression (0.1 mg/kg per HLH-2004 guidelines (31)), Dexamethasone tapered to 3 mg (23–28 May), then 1.5 mg from 29 May.
	IV Immunoglobulin (IVIG)	20 g daily	16–20 May 2023	Immunomodulation
Supportive Care	Dopamine + Metaraminol		12 May 2023	Management of septic shock, titrated to maintain MAP >65 mmHg
	Avatrombopag	60 mg daily	18–23 May 2023	Thrombocytopenia
	Granulocyte Colony-Stimulating Factor (G-CSF)	150 µg single dose	12 May 2023	Neutropenia
	Low Molecular Weight Heparin (LMWH)	5000 IU daily	16–22 May 2023	Coagulopathy prophylaxis
	Human Albumin	12.5 g daily	15–24 May 2023	Hypoalbuminemia support
Hepatoprotectants	Magnesium Isoglycyrrhizinate	200 mg daily	12–29 May 2023	Liver support
	Glutathione	1800 mg daily	12–29 May 2023	Liver support
	Adenosylmethionine (SAMe)	1.0 g daily	12–29 May 2023	Liver support

The patient’s body weight was 50 kg. Dosing was dynamically adjusted based on hepatic function, guideline recommendations, and the need for targeted therapy (e.g., spinal penetration).

TB, tuberculosis; HLH, hemophagocytic lymphohistiocytosis; IV, intravenous; INH, Isoniazid; RIF, Rifampicin; EMB, Ethambutol; LZD, Linezolid; DEX, Dexamethasone; IVIG, Intravenous Immunoglobulin; G-CSF, Granulocyte Colony-Stimulating Factor; LMWH, Low Molecular Weight Heparin; SAMe, S-Adenosylmethionine.

### 2.2 Imaging evolution

18F-fluorodeoxyglucose positron emission tomography/computed tomography (PET-CT) on day 8 showed FDG-avid T9 lesions and hepatosplenomegaly (**Figures 1C, D**). Magnetic resonance imaging (MRI) on day 10 confirmed tuberculous spondylitis with a paraspinal abscess (**Figures 1E, F**). Bone marrow biopsy performed on day 7 concurrently revealed hemophagocytosis (**Figures 1G, H**), supporting HLH. Follow-up CT on day 14 revealed partial resolution of the miliary nodules and a reduction in the pleural effusions (**Figure 1B**). CT-guided T9 biopsy (day 16) showed caseating granulomas, and next-generation sequencing (NGS) identified Mycobacterium tuberculosis complex.

### 2.3 Treatment adjustment and outcome

Following the improvement of liver function (ALT <80 U/L, total bilirubin <2×ULN) and lymphocyte count recovery (ALC 1.10×10^9^/L) by day 14, a tailored oral anti-TB regimen was initiated: isoniazid (0.3 g daily), rifampicin (0.45 g daily), ethambutol (0.75 g daily), moxifloxacin (0.4 g daily), and linezolid (0.6 g twice daily). Dexamethasone and intravenous immunoglobulin were continued. The patient’s condition improved markedly: defervescence occurred within 72 hours, renal function recovered by day 4, platelet counts normalized by day 11, and inflammatory markers declined steadily. Upon clinical stabilization, he was discharged on 29 May to continue this optimized oral regimen. Follow-up imaging revealed resolution of miliary nodules on chest CT at one month and a >50% reduction in the spinal abscess on MRI at three months (**Supplementary Table S2**).

## 3 Discussion

### 3.1 Clinical trajectory, diagnostic complexities, and the adult TB-HLH landscape

Tuberculosis-associated hemophagocytic lymphohistiocytosis (TB-HLH) is a rare, often fatal adult condition. While literature is largely pediatric, adult management data remain limited (3, 5). Systematic reviews confirm high mortality (30–50%), with advanced age as a key risk factor (6, 7); our 60-year-old patient thus represented a high-risk presentation. Despite antibiotics, he developed progressive cytopenias, coagulopathy, hyperferritinemia, and multi-organ dysfunction, signaling overt HLH with shock physiology (8). His peak ferritin (5,802 ng/mL) was lower than the >10,000 ng/mL often seen in TB-HLH (3, 9), underscoring that diagnosis cannot rely on this parameter alone. Markedly elevated ferritin, CRP, and proinflammatory cytokines (IL-6, IFN-γ), along with rapid defervescence after steroids, established a profound cytokine storm (8, 10). Diagnostic challenges included an initially negative T-SPOT.TB (11) and the initial use of empiric therapy for atypical pathogens (including omadacycline (12)), which lacked efficacy against M. tuberculosis and may have compromised microbiological yield, delaying diagnosis. Critically, T9 spondylitis was an occult driver of dissemination, with vertebral NGS providing the definitive, culture-negative diagnosis (13).

### 3.2 Mechanisms of TB-associated cytokine storm: insights from mycobacterial HLH

This case exemplifies how disseminated tuberculosis triggers secondary HLH and cytokine storm. The spinal and miliary pulmonary foci served as synergistic antigen reservoirs, sustaining T-cell and macrophage activation with excessive production of IFN-γ, IL-1β, IL-6, IL-18, and TNF-α (8, 14). Recent studies highlight a dominant IFN-γ/IL-18 signature in mycobacterial HLH, sometimes with underlying genetic variants in HLH-related genes (e.g., PRF1, UNC13D) that lower the disease threshold (15, 16). Inflammasome activation (e.g., NLRP3, AIM2) in TB drives IL-1β and IL-18 maturation, amplifying the cytokine storm (17, 18). The IL-18–driven dysregulation of IFN-γ, which forms a self-amplifying loop in TB-HLH (6), is a key mechanism leading to T-cell exhaustion, a state characterized by impaired effector function (19). In disseminated TB, high antigen load drives concurrent T-cell exhaustion and hyperinflammation, impairing pathogen clearance while sustaining inflammation (20, 21). Thus, the spinal lesion was a key immunopathological driver, illustrating a core mechanism of TB-associated cytokine storm.

### 3.3 Host predisposition: immunosenescence and risk factors

The patient’s advanced age constituted a significant risk factor, aligning with systematic reviews that identify older age as a key determinant of mortality in TB-HLH (6, 7). Although no overt immunodeficiency was diagnosed, the converging effects of immunosenescence and a substantial smoking history compromised immune containment, thereby predisposing to disseminated disease (1, 22–24). In this context, the profound CD4^+^ T-cell lymphocytopenia likely stemmed from the combined insults of disseminated TB, HLH, and septic shock, mediated by mechanisms including activation-induced cell death, hyperinflammatory cytotoxicity, and sepsis-induced immunoparalysis (21, 25, 26). Immunosenescence is implicated in both facilitating the initial hematogenous dissemination and lowering the threshold for a fulminant cytokine storm (22), while chronic smoking acted synergistically to exacerbate immune dysregulation and impair mucosal barrier integrity (23, 24). Although undiagnosed hypomorphic genetic variants in genes such as PRF1 or UNC13D remain a theoretical predisposition (16, 27), the patient’s rapid response to therapy strongly supports a predominant, infection-driven secondary etiology.

### 3.4 Therapeutic strategy and rationale

Following initial stabilization for septic shock, management centered on a dual-pathway strategy addressing both disseminated tuberculosis and secondary HLH. Significant hepatic dysfunction initially prompted a liver-sparing anti-TB regimen (moxifloxacin, amikacin) (28). Upon hepatic recovery, therapy transitioned to a standard oral regimen, augmented with linezolid for bone penetration (29, 30). Concurrently, immunomodulation with dexamethasone was initiated per guidelines for HLH (31, 32), with adjunctive IVIG for the fulminant cytokine storm despite limited evidence in adult TB-HLH (33). For steroid-refractory HLH, guidelines support considering IL-1/IL-6 blockade (32), whereas anti–IFN-γ therapy (emapalumab) experience in TB is limited (34). Future immunoprofiling may guide therapy (35). The patient’s rapid improvement underscores this combined approach’s value. Throughout his treatment and subsequent follow-up, the patient remained grateful for his care, reporting a significantly improved quality of life and commitment to completing anti-tuberculous therapy.

### 3.5 Strengths and limitations

This report’s strengths include a comprehensive diagnostic workup and successful dual-pathway management in a high-risk host. Limitations include the single-case design and the immunologic scope. Although we documented CD4^+^ lymphocytopenia and a cytokine storm, deeper immunophenotyping and genetic studies could have better elucidated the immunopathology.

In conclusion, disseminated TB, even from focal spondylitis without overt immunodeficiency, can trigger fulminant HLH. In TB-endemic areas, clinicians should investigate for disseminated TB in patients with hyperinflammation and start early combined anti-TB and immunomodulatory therapy to improve outcomes.

## Data Availability

The original contributions presented in the study are included in the article/**Supplementary Material**. Further inquiries can be directed to the corresponding authors.

## Glossary

ALT: Alanine aminotransferase
ALC: Absolute lymphocyte count
AST: Aspartate aminotransferase
CRP: C-reactive protein
CT: Computed tomography
DB: Direct bilirubin
DEX: Dexamethasone
EBV: Epstein-Barr virus
EMB: Ethambutol
FDG: Fluorodeoxyglucose
G-CSF: Granulocyte colony-stimulating factor
Hb: Hemoglobin
HLH: Hemophagocytic lymphohistiocytosis
IFN-γ: Interferon-gamma
IL-1β: Interleukin-1 beta
IL-6: Interleukin-6
IL-18: Interleukin-18
INH: Isoniazid
IV: Intravenous
IVIG: Intravenous immunoglobulin
LAC: Lactate
LMWH: Low molecular weight heparin
LZD: Linezolid
MRI: Magnetic resonance imaging
NGS: Next-generation sequencing
PET-CT: Positron emission tomography/computed tomography
PLT: Platelet
RIF: Rifampicin
SAMe: S-adenosylmethionine
SCrea: Serum Creatinine
SOFA: Sequential Organ Failure Assessment
SUVmax: Maximum standardized uptake value
TB: Tuberculosis
T-Bil: Total bilirubin
TNF-α: Tumor necrosis factor-alpha
ULN: Upper limit of normal
WBC: White blood cell count
