# Supplementary material for: Tuberculosis-triggered cytokine storm with hemophagocytic lymphohistiocytosis and tuberculous spondylitis in an apparently immunocompetent host: a case report and literature review
Source: Front Immunol. 2025 Nov 21;16:1695605. doi: 10.3389/fimmu.2025.1695605 (PMC12678318; doi:10.3389/fimmu.2025.1695605)
Supplement: Supplementary file 1 [file Table1.docx]

****Supplementary Table S1. Comprehensive Diagnostic Evaluation for Hemophagocytic Lymphohistiocytosis (HLH)****

|  | **Diagnostic Parameter** | **Finding in This Case** | **HLH-2004 Criterion Met** | **HScore Assignment** |
| --- | --- | --- | --- | --- |
| 1 | Fever | Present (>38.5°C) | Yes | +33 (Temperature ≥ 38.4 °C) |
| 2 | Splenomegaly | Present (confirmed by PET-CT) | Yes | +38 (Hepatomegaly and/or splenomegaly) |
| 3 | Cytopenias (≥2 lineages) | Affecting 3 lineages (WBC, Hb, PLT) | Yes | +34 (3 lineages) |
| 4 | Hypertriglyceridemia & Hypofibrinogenemia | TG: 4.1 mmol/L; Fibrinogen: 1.2 g/L | Yes | +44 (TG ≥1.5 mmol/L); +24 (Fibrinogen ≤2.5 g/L) |
| 5 | Hemophagocytosis | Present in bone marrow | Yes | +35 (Hemophagocytosis on marrow aspirate) |
| 6 | Hyperferritinemia | 5,802 ng/mL | Yes | +50 (Ferritin ≥2000 µg/L) |
| 7 | Soluble IL-2 Receptor | Not Tested | Not Assessed | (Not a parameter) |
| 8 | NK-cell Activity | Not Tested | Not Assessed | (Not a parameter) |
|  | Additional HScore Parameters |  |  |  |
|  | Known Immunosuppression | No | (Not a criterion) | 0 |
|  | AST Level | Elevated (Peak: 213 U/L) | (Not a criterion) | 0 (No known immunosuppression) |
|  |  |  |  |  |
|  |  | **HLH-2004 Diagnostic Summary:** | **5 out of 8 Criteria Met** | **→ Diagnosis Confirmed** |
|  |  | **HScore Summary:** | **Total Score: 258** | **→ Probability of HLH: >99%** |

**Note:** The HLH-2004 diagnostic and therapeutic guidelines require meeting 5 out of 8 criteria [31]. The HScore ([https://saintantoine.aphp.fr/score/](https://saintantoine.aphp.fr/score/)) is a complementary diagnostic tool, where a score of ≥250 corresponds to a >99% probability of HLH [4]. Although the patient's AST was elevated, it contributes no points to the HScore in the absence of a known immunocompromised state. Splenomegaly was confirmed by imaging, which is sufficient to fulfill the criterion.
Abbreviations: TG, triglycerides.
