# Supplementary material for: Tuberculosis-triggered cytokine storm with hemophagocytic lymphohistiocytosis and tuberculous spondylitis in an apparently immunocompetent host: a case report and literature review
Source: Front Immunol. 2025 Nov 21;16:1695605. doi: 10.3389/fimmu.2025.1695605 (PMC12678318; doi:10.3389/fimmu.2025.1695605)
Supplement: Supplementary file 2 [file Table2.docx]

****Supplementary Table S2. Comprehensive Diagnostic and Therapeutic Chronology in Disseminated Tuberculosis Complicated by Hemophagocytic Lymphohistiocytosis****

| **Time Point** | **Diagnostic Milestones** | **Therapeutic Interventions** | **Clinical Trajectory & Outcomes** |
| --- | --- | --- | --- |
| **Day 1** | • **Radiologic suspicion**: Chest CT demonstrates miliary nodules and pleural effusions, raising initial concern for disseminated tuberculosis  • **Laboratory evidence**: Pancytopenia (WBC 1.75×10⁹/L, platelets 71×10⁹/L), marked lymphopenia (ALC 0.12×10⁹/L), hyperferritinemia (2,521 ng/mL), cholestatic hepatitis (total bilirubin 86.9 μmol/L), renal impairment (SCrea, 126 μmol/L)  • **Septic shock:** lactic acidosis (LAC 3.3 mmol/L) with compensatory respiratory alkalosis (pCO₂ 26.5 mmHg). | • **Supportive care**: fluid resuscitation and vasopressor support for Septic shock  • **Empiric therapy**: Omadacycline initiated for potential atypical pneumonia | • **Presentation**: Febrile (38.1°C) with syncopal episodes  • **Physical findings**: Hypotension (80/40 mmHg), icteric sclera and skin  • **Clinical status**: No palpable lymphadenopathy or hepatosplenomegaly |
| **Day 2** | • **Progressive cytopenias**: Worsening hematologic parameters | • **Continued support**: Maintenance of empiric antibiotic coverage and hemodynamic support | • **Clinical deterioration**: Ongoing fever and constitutional symptoms |
| **Day 4** | ****• Cellular immunodeficiency:**** Flow cytometry reveals profoundly depleted CD4^+^ T-cells (213 cells/μL) and CD8^+^ T-cells (145 cells/μL)  • ****Partial lymphocyte recovery:**** ALC increases to 0.87×10⁹/L  • **Syndrome confirmation**: HLH-2004 diagnostic criteria fulfilled with HScore 258 (>99% probability)  • **Microbiological support**: Repeat T-SPOT.TB is positive  • **Inflammatory surge:** Characterized by a ferritin peak of 5,802 ng/mL, elevated inflammatory markers (CRP 125.3 mg/L, IL-6 22.47 pg/mL, IFN-γ 10.5 pg/mL), hypertriglyceridemia (4.1 mmol/L), and hypofibrinogenemia (1.2 g/L). | **• None** (immunomodulation not yet started) | • **Hematologic crisis**: Platelet nadir (18×10⁹/L)  • **Systemic inflammation**: Marked elevation of inflammatory markers |
| **Day 5** | **• None additional** | **• Immunomodulation initiation:** Dexamethasone (0.1 mg/kg/day, 5 mg daily) and intravenous immunoglobulin initiated for HLH management  **• Targeted antimycobacterial therapy:** Liver-sparing regimen initiated (moxifloxacin 0.4g daily)  **• Sepsis coverage:** Meropenem continued for empirical bacterial coverage | • **Critical status**: Persistent cytopenias and systemic inflammation |
| **Day 7** | • **Histopathologic confirmation**: Bone marrow biopsy demonstrates definitive hemophagocytosis | • **Supportive management:** Avatrombopag initiated for persistent thrombocytopenia  • **Antimycobacterial therapy:** Amikacin added (400 mg daily) | • **Stable critical condition**: Ongoing management in intensive care setting |
| **Day 8** | • **Metabolic imaging**: PET/CT reveals FDG-avid T9 vertebral lesion (SUVmax 8.4) with hepatosplenomegaly | • **Therapeutic continuation**: Maintenance of dual-pathway strategy | • **Early response**: Initial signs of inflammatory marker improvement |
| **Day 10** | • **Anatomic characterization**: MRI confirms tuberculous spondylodiscitis with paraspinal abscess formation | • **Regimen optimization**: Continued immunomodulation and antimicrobial therapy | • **Stabilizing trend**: Gradual improvement in clinical parameters |
| **Day 11** | • **Renal function recovery:** Serum creatinine normalizes (72 μmol/L) |  | • **Hematologic recovery**: Platelet count normalizes, indicating initial treatment response |
| **Day 14** | **• Immune reconstitution:** ALC returns to low-normal range (1.10×10⁹/L)  • **Hepatic recovery**: Liver function improves (ALT <80 U/L, total bilirubin <2×ULN)  • **Inflammatory resolution**: CRP demonstrates significant decline (9.5 mg/L) | • **Therapeutic escalation**: Introduction of oral first-line anti-tuberculous therapy (isoniazid 0.3 g daily, rifampicin 0.45 g daily, ethambutol 0.75 g daily, moxifloxacin 0.4 g daily, and linezolid 0.6 g twice daily) | • **Clinical improvement**: Afebrile with resolving cytopenias and inflammatory markers |
| **Day 16** | • **Microbiological confirmation**: CT-guided T9 biopsy shows caseating granulomas; next-generation sequencing identifies Mycobacterium tuberculosis complex without resistance mutations | • **Diagnosis confirmation**: Definitive microbiological diagnosis enables targeted therapy continuation | • **Consolidated improvement**: Sustained clinical and laboratory improvement |
| **Day 18** | • **Discharge readiness**: Clinical stability achieved with normalized hematologic and inflammatory parameters | • **Regimen consolidation**: Discharge on the optimized oral anti-tuberculous regimen. | • **Successful outcome**: Clinical stabilization permitting hospital discharge |
| **1-month follow-up** | • **Radiologic resolution**: Chest CT shows complete resolution of miliary nodules | • **Outpatient management**: Continuation of consolidated anti-tuberculous regimen | • **Sustained recovery**: Asymptomatic with normal functional status |
| **3-month follow-up** | • **Structural improvement**: MRI demonstrates >50% reduction in spinal abscess size | • **Long-term therapy**: Ongoing antimycobacterial treatment with excellent adherence | • **Durable response**: Maintained clinical remission with radiologic improvement |

**Abbreviations**: HLH, hemophagocytic lymphohistiocytosis; TB, tuberculosis; CT, computed tomography; PET, positron emission tomography; MRI, magnetic resonance imaging; NGS, next-generation sequencing; IVIG, intravenous immunoglobulin; ALT, alanine aminotransferase; CRP, C-reactive protein; WBC, white blood cell count; ALC, absolute lymphocyte count; LAC, lactate; IL-6, interleukin-6; IFN-γ, interferon-gamma; SUVmax, standardized uptake value maximum; SCrea, creatinine.
